# Nurses’ Interventions in Minimizing Adult Patient Vulnerability During Extracorporeal Membrane Oxygenation as a Bridge to Lung Transplantation: An Integrative Review

**DOI:** 10.1177/23779608241262651

**Published:** 2024-07-26

**Authors:** Nuno Costa, Helga Rafael Henriques, Candida Durao

**Affiliations:** 1 Unidade Local de Saúde São José – Hospital de São José, Lisboa, Portugal; 2 86712 Escola Superior de Enfermagem de Lisboa, Lisboa, Portugal; 3 Escola Superior de Enfermagem de Lisboa; CIDNUR – Nursing Research, Innovation and Development Centre of Lisbon, Lisboa, Portugal

**Keywords:** critical care nursing, critically ill, extracorporeal membrane oxygenation, lung transplantation, vulnerability

## Abstract

**Introduction:**

People during extracorporeal membrane oxygenation (ECMO) as a bridge to lung transplantation find themselves in a high degree of physical and psychological vulnerability, which could cause additional problems for their health status. Therefore, this review aims to identify the interventions that shape critical nursing care to minimize patient vulnerability during ECMO as a bridge to lung transplantation.

**Method:**

A literature review was performed using CINAHL, MEDLINE, PubMed, Scopus and Web of Science databases with searches conducted in March 2023, with temporal restriction of articles published between 2013 and 2023. After selecting articles involving adults in critical situations on ECMO, their quality was assessed using the critical appraisal tools from the Joanna Briggs Institute. Articles with the pediatric population, reviews, and opinion articles were excluded. A spreadsheet was built for data extraction and a narrative analysis was performed.

**Results:**

Three articles were included involving 40 participants in total. Interventions that shape critical nursing care to minimize a person's vulnerability are in the physical domain (basic precautions to prevent infection) and in the psychological domain (trusting relationships, consistent and clear communication, physical presence of nurses and family members and the use of advocacy). The Awake ECMO strategy was identified as beneficial for reducing vulnerability.

**Conclusion:**

By recognizing and identifying the person's vulnerability during ECMO as a bridge to lung transplantation, nurses can implement effective interventions to minimize vulnerability in this population, thus contributing to the person's well-being through personalization and individualization of care. Additionally, the results of this review could be useful for developing tools to assess the degree of vulnerability and for implementing person-centered care measures and policies. However, further research is warranted given the scarcity of literature on these topics.

## Introduction

For many people with end-stage lung disease, transplantation is the last available treatment capable of offering a better quality of life and, in more severe cases, sustaining life (Kearns & Hernandez, 2016; Koons & Siebert, 2020). However, it is observed that the number of people on the waiting list for lung transplants exceeds the number of organs available for donation (Faccioli et al., 2021; Kearns & Hernandez, 2016). Since 2019, the year in which the highest number of lung transplants were performed (*n* = 2,759), the number has been decreasing, with only 2,569 transplants performed in 2021. Despite this number, there were 4,117 candidates on the waiting list at the end of the year, and a waitlist mortality rate of 17.6% (Valapour et al., 2023). These data induce great anxiety in transplant candidates.

Due to this reality, and to reduce the mortality rate of people who are on the waitlist for lung transplant and present an abrupt deterioration of their end-stage lung disease, extracorporeal membrane oxygenation (ECMO) has been increasingly used as a bridge to lung transplantation (Hayanga et al., 2019; Loor et al., 2021). ECMO's ability to replace the function of the lungs or heart for prolonged periods allows it to be used as a bridge to transplantation in cases of end-stage respiratory or heart failure, a bridge to recovery in cases of acute reversible illness, and a bridge for decision-making when the prognosis remains uncertain (Abrams et al., 2014).

The publication of the CESAR study in 2009 redefined the therapeutic approach in adults with respiratory failure, contributing to the widespread use of ECMO (Peek et al., 2009). That same year saw significant growth in the use of ECMO internationally, associated with technological advances in the technique and the emergence of the H1N1 pandemic (Rawal et al., 2017). More recently, ECMO was widely used for acute respiratory distress syndrome related to coronavirus disease 2019 (Ling et al., 2022). The use of ECMO as a bridge to lung transplantation can enable patients to breathe spontaneously, participate in physical therapy, eat and drink normally, preventing invasive mechanical ventilation (IMV), sedative drugs, and associated risks such as pulmonary infections, immobility, and delirium (Diaz-Guzman et al., 2013; Kearns & Hernandez, 2016). Furthermore, the survival of patients with ECMO as a bridge to lung transplant has improved, with some centers reporting better outcomes than mechanical ventilation (Hayanga et al., 2020).

Despite the benefits, there is an incidence of complications related to the use of ECMO support due to its invasive nature, such as significant bleeding, cannula dislodgement, hemolysis, thrombotic events, bloodstream infection, or renal dysfunction, which may result in removal from the transplant waitlist and contribute to more significant mortality and morbidity (Kearns & Hernandez, 2016; Kim et al., 2022). On the other hand, people are necessarily admitted and confined to the intensive care unit (Abrams et al., 2014) and its technological and impersonal environment, with consequences for the patient and family.

The transplantation is usually prompted by patients with diagnoses of end-stage lung diseases, including pulmonary hypertension, bronchiectasis, pulmonary fibrosis, and pulmonary emphysema (Pola-Dos-reis et al., 2020). These complex chronic diseases require a comprehensive and specialized approach for proper management and treatment. People with at least one or more chronic diseases and comorbidities, and who consume a very high level of health resources, such as those with chronic lung disease, are defined as complex chronic patients (Vargiu et al., 2017).

According to Chesnay (2019), chronically ill patients are vulnerable. When these people have an acute exacerbation of their underlying lung disease, they become critically ill patients, usually requiring intensive care and technology to preserve life. In these situations, patients are highly vulnerable due to the significant dependence and severity of the situation, feeling unprotected and highly susceptible to risks and damages (Mckinley et al., 2002).

Vulnerability has a specific connotation in health care, meaning susceptibility/risk to health problems (Chesnay, 2019). Purdy (2004) defines the concept as a highly individualized dynamic process where circumstances influence the results. It is a condition inherent to human beings that accompanies them throughout their lives and cannot be eliminated or overcome (Morais & Monteiro, 2017), where the vulnerability degree of each person is defined and influenced by personal and environmental resources, according to Rogers’ vulnerability model (Rogers, 1997).

Three main vulnerability domains emerge from the literature: physical, psychological, and social (Scanlon & Lee, 2007). Physical vulnerability refers to a person's greater susceptibility to suffering more damage and the inability to resist further harm caused by a weakened disease state, leading to more significant morbidity and mortality. Psychological vulnerability refers to emotional effects caused by disease and treatment, potentially damaging the person's identity, autonomy, and self-esteem (Cousley, 2015; Scanlon & Lee, 2007).

Although evidence in this area is scarce, it is assumed that people during ECMO as a bridge to lung transplantation are at a higher vulnerability degree in the physical domain, related to disease progression, the potential complications associated with the use of ECMO, and the ICU environment. Also, in the psychological domain, the patients awaiting lung transplantation have a high incidence of anxiety and panic disorders (Parekh et al., 2003). According to Koons and Siebert (2020), waiting for a transplant and the need for ECMO support is accompanied by fear and stress, with fear being an expression of vulnerability (Boykin & Schoenhofer, 2013).

Being vulnerable is a phenomenon that challenges nursing practice (Purdy, 2004). Nursing addresses human vulnerability due to its implications for people's health and, consequently, the need to identify healthcare needs to ensure their protection (Nichiata et al., 2008). The study's significant finding of McKinley et al. (2002) was that vulnerability better represented the experiences of critically ill patients in intensive care. Therefore, nurses can positively influence the patient's experience and outcomes when they better understand and recognize patients’ vulnerability.

Given the above, this review aims to identify the interventions that shape critical nursing care to minimize patient vulnerability during ECMO as a bridge to lung transplantation.

## Method

An integrative literature review approach was followed, using the six steps defined by Toronto and Remington (2020) to guide the design: (1) formulate review question; (2) search and select literature systematically; (3) quality appraisal; (4) analysis and synthesis; (5) discussion and conclusion; and (6) dissemination.

This methodological approach was selected as it allows to answer questions about nursing, guiding nursing practice through conducting a comprehensive literature search and revealing gaps in knowledge that suggest further studies to be undertaken (Toronto & Remington, 2020). According to the same authors, the integrative review process involves: identifying a problem, formulating review questions, defining eligibility criteria (Step 1); systematic literature search and selection (Step 2); assessing article quality (Step 3); data extraction and analysis (Step 4); reflecting on findings and drawing conclusions (Step 5).

### Formulate Review Question

A starting question was formulated according to the PI[C]O structure (Population (P)/Intervention (I)/[Comparison (C)]/Outcome (O); Aromataris & Munn, 2020). The question was: Which are the interventions that shape critical nursing care (I) to minimize patient vulnerability (O) during ECMO as a bridge to lung transplantation (P)?

### Search and Select Literature Systematically

The search for literature was carried out in March 2023. CINAHL (Cumulative Index to Nursing and Allied Health Literature; EBSCOhost), MEDLINE (EBSCOhost), PubMed, Scopus and Web of Science were electronically searched. The search encompassed articles published between January 2013 and May 2023. The search terms used for this review were: “critical illness,” “critically ill patients,” “extracorporeal membrane oxygenation,” “ECMO,” “critical care nursing,” “nursing care,” “nursing interventions,” “treatment,” “interventions,” “vulnerability,” “psychological well-being,” “life experiences,” “signification,” “meaning,” “experience,” “life purpose,” and “attitude to life”. To increase the precision and accuracy of the results, we used Boolean operators such as “and” and “or.” The same search strategy (Table 1) was used in all databases.

**Table 1.** Search Strategy.

((“Critical illness”) OR (“critically ill patients”)) AND ((“extracorporeal membrane oxygenation”) OR (ECMO))
AND
((“critical care nursing”) OR (“nursing care”) OR (“nursing interventions”) OR (treatment) OR (interventions))
AND
((Vulnerability) OR (“Psychological Well-Being”) OR (“Life experiences”) OR (Signification) OR (Meaning) OR (Experience) OR (“Life purpose”) OR (“Attitude to Life”))

The inclusion criteria were: (a) adults in critical situations on ECMO; (b) patients with age equal to or greater than 18 years; (c) critical patients admitted to the ICU, and (d) original articles, to collect more detailed and specific data relevant to the aim of the integrative review. Studies with a publication date before 2013, articles with the pediatric population, letters and opinion articles were excluded. We excluded review articles from our review because our focus was on examining the results and findings of original studies related to our research objective. We chose to prioritize the investigation of primary studies to provide a more direct and specific analysis of the topic at hand. For the same reason, articles with incomplete text, such as abstracts and conference proceedings, were also an exclusion criterion during the selection process. We had access to the full text of the identified articles. The studies analyzed were in English, Portuguese, and Spanish to ensure a good quality selection procedure and data extraction.

**Figure 1.**
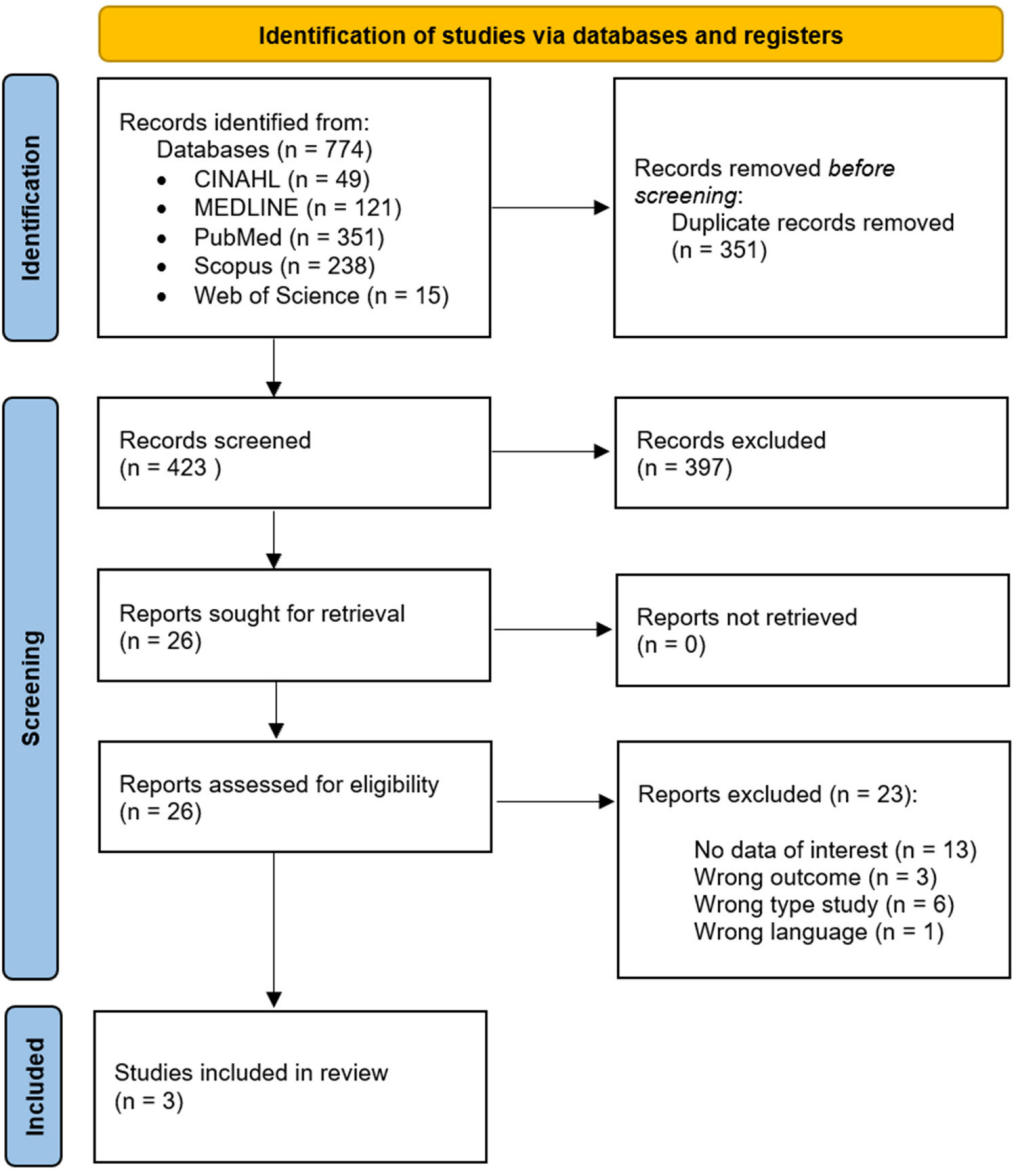
PRISMA Flow Diagram (Page et al., 2021).

The search in the databases resulted in 774 articles. All articles were imported into Rayyan (Ouzzani et al., 2016), where 351 duplicate articles were removed before the screening, leaving 423 articles. The three reviewers followed the article's process selection blindly and independently, considering the defined criteria. The researchers screened titles and abstracts in the Rayyan application (Ouzzani et al., 2016), and the entire reading of the articles was then undertaken. The article selection process that gave rise to this integrative review is shown in Figure 1 according to the Preferred Reporting for Systematic Reviews and Meta-Analysis Flow Diagram 2020 (Page et al., 2021). The results followed the identification, screening, and inclusion process, resulting in three articles. All disagreements and conflicts related to the selection process and the inclusion of articles were resolved through discussion.

PRISMA = Preferred Reporting for Systematic Reviews and Meta-Analysis.

The integrative review protocol is registered in the international database PROSPERO with the ID CRD42023432481. Its title has been modified to ensure better alignment.

### Quality Appraisal

For the article's methodological quality evaluation, critical appraisal tools from the Joanna Briggs Institute were used to ensure the assessment of reliability, relevance, and results of selected articles (Moola et al., 2020), independently by the researchers. The risk of bias disagreements was mitigated through discussion.

### Analysis and Synthesis

The reviewers obtained data from the articles, using a spreadsheet built for data management, including author, year, and country; study design; aim; participants; limitations; critical appraisal; vulnerability factors; interventions; and outcomes/findings. After the data collection, a narrative analysis was performed based on Rogers’ vulnerability model. A graphical representation of the model was used to better understand the vulnerability.

## Results

The bibliographical sample of this review consists of three articles that meet the previously defined eligibility criteria and allow the answer to the research question. All articles are primary studies, two of which are quantitative studies (one retrospective cohort study and one case series), and one is a qualitative approach through interpretive description. The included studies took place in three different countries: Italy (*n* = 1), Turkey (*n* = 1), and the United States of America (*n* = 1). Physicians carried out the two quantitative studies, while nurses carried out the qualitative study. All studies considered were of high quality with a cutoff score of higher than 70% on the critical appraisal tools, according to Joanna Briggs Institute Critical Appraisal Tools (Moola et al., 2020). However, the cohort study fails to state strategies to deal with confounding factors, while the statistical analysis carried out in the case series study could be more extensive and accurate. On the other hand, in the qualitative study, it is unclear whether the researcher's influence on the research and vice versa is addressed.

A presentation and data analysis are performed in Table 2, conducted as described in the methodology to understand the included studies better.

**Table 2.** Systematization of Article Findings Included in the Integrative Review.

Author, year, and country	Study design	Aim	Participants	Limitations	Critical appraisal	Vulnerability factors	Interventions	Outcomes/findings
Nosotti et al., 2013 (Italy)	Retrospective cohort study	Review practice in bridging critical candidates to lung transplantation with extracorporeal membrane oxygenation (ECMO), by comparing patients with IMV with patients with spontaneous breathing.	*n *= 11 patients underwent lung transplantation after ECMO support: “Awake-ECMO” group (*n *= 7) IMV-ECMO group (*n *= 4)	Single center, nonrandomized study. Small number of patients.	9/11	The use of systemic anticoagulation increases blood complications, and patients who present bleeding problems tend to have a longer ECMO bridge time. Use of sedation and IMV.	Awake ECMO	Participation in clinical progression; Spontaneous feeding; Possibility of physiotherapy; Interaction with family members and a multidisciplinary team; Prevention of complications associated with invasive mechanical ventilation; Less time on IMV, hospitalization in an intensive care unit and hospitalization after transplantation; One-year survival rate of 85.7% in spontaneously breathing patients versus 50% in patients with IMV.
Vayvada et al., 2021 (Turkey)	Retrospective study—case series	Present initial experience with the use of ECMO as a bridge to lung transplant	*n *= 13 patients underwent ECMO as a bridge to lung transplantation —Successful bridging (*n *= 7) —Failed bridging (*n *= 6)	Noninclusion of patients who were receiving ECMO but were not included on the waiting list. Limited sample size.	8/10	Development of nosocomial infections related to ECMO. Use of ECMO as a bridge to lung transplantation is associated with increased primary graft dysfunction and prolonged hospitalization.	Awake ECMO Infection prevention	Awake ECMO prevents the use of sedation and IMV, facilitating early and active mobilization; Enabling oral feeding and drinking.
Knudson et al., 2022 (United States)	Single qualitative study of interpretive description	Describe the experiences and needs of adults treated with ECMO, from the onset of illness symptoms through the process of survivorship	*n *= 16 adults survivors of ECMO	Most participants tended to be reasonably healthy prior to ECMO illnesses, which may limit the transferability of findings. Different recruitment strategies, which increase the risk of potential selection bias.	8/10	High morbidity rates associated with the use of ECMO. Risk of infection, bleeding, ischemia. Deficiencies in communication, knowledge, and comprehension. Feelings of loneliness. Limited ability to advocate. Pain and discomfort. Sleep disorders.	Awake ECMO Presence of family Establishing a trusting relationship by communicating consistent and clear information compassionately and appropriately. Knowing people as people and moment-to-moment, providing enough information to empower not overwhelm. Advocacy.	The use of awake ECMO allowed the person to maintain autonomy and identity; The physical presence of the family led to decreased feelings of loneliness, increased peace of mind and better advocacy on behalf of survivors.

IMV = invasive mechanical ventilation.

The results of this review allowed us to identify major findings that shape critical nursing care in minimizing people's vulnerability during ECMO as a bridge to lung transplantation in the physical domain: use of essential infection prevention and control measures (Vayvada et al., 2021); in the psychological domain: the presence of family, use of advocacy, and the establishment of trusting relationships, through gauging the person's individual care needs, recognizing their individuality and uniqueness (Knudson et al., 2022); and in both domains: Awake ECMO strategy, as it allows the person to maintain his autonomy and identity (Knudson et al., 2022), and prevents people deterioration, contributing to a better condition pretransplant, and consequently, a better posttransplant outcome (Nosotti et al., 2013; Vayvada et al., 2021).

**Figure 2.**
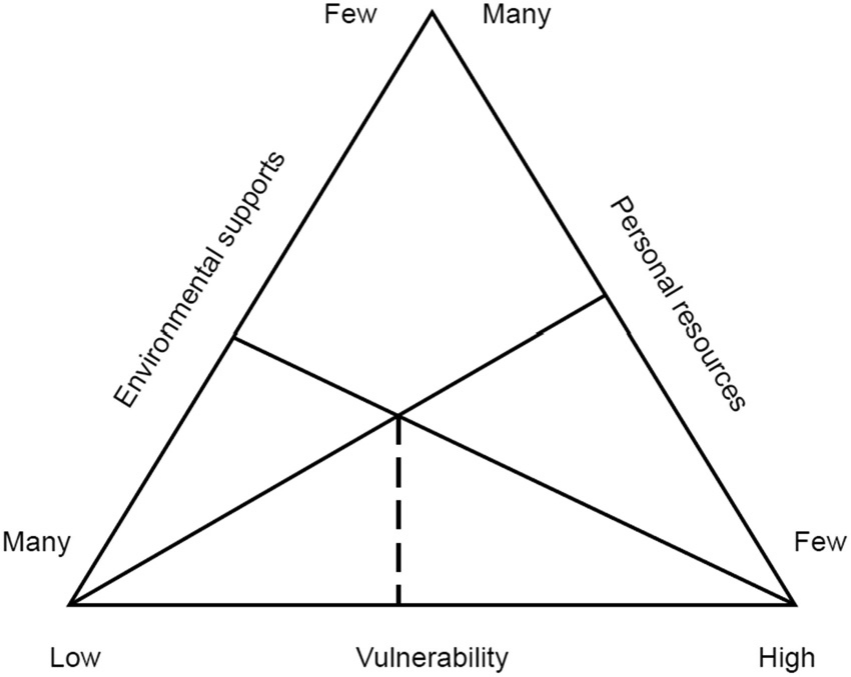
Rogers’ Vulnerability Model (Rogers, 1997).

These interventions will contribute to increasing the patient's personal resources and environmental support, thus reducing their vulnerability according to Rogers’ Vulnerability Model (Figure 2).

## Discussion

When patients are admitted to the hospital, they experience some form of vulnerability, which, if not properly addressed by nurses, can lead to further health problems and negative outcomes (Scanlon & Lee, 2007). In this sense, critical care nurses should enhance their knowledge of the factors/predictors of vulnerability and the appropriate interventions to mitigate these experiences.

The heightened level of physical and emotional dependency experienced by critically ill individuals in the ICU renders them vulnerable, compounded by factors such as difficulty in communicating, depersonalizing care, lack of knowledge about the condition, feelings of loneliness, sleep disorders, the presence of pain, discomfort, and other frightening physical experiences, namely the fear of dying (McKinley et al., 2002). The greater susceptibility to occurrences of damage associated with the identified factors implies that nursing care is guided and based on surveillance. Surveillance has nursing knowledge at its core, being a prerequisite for informed and intentional nursing care (Meyer & Lavin, 2005). Asserting itself as a central function of nurses, vigilance plays a prominent role in the safety of care (Benner, 2001), and consequently, in reducing vulnerability.

The risk of infection, bleeding, and vascular complications resulting from cannulation in the person during ECMO as a bridge to lung transplantation were identified as vulnerability factors in the physical domain The selected articles identified basic precautions to prevent infection as a nursing intervention in minimizing physical vulnerability (Vayvada et al., 2021).

Preventing and controlling healthcare-associated infections is fundamental in nursing practice, being one of Florence Nightingale's many healthcare reforms, known as Environmental Theory, which still survives to this day (Gilbert, 2020). It is a way of defending the patient's best interest, as the appearance of opportunistic diseases can affect and infect the weakened individual, further reducing their capacity to recover (Scanlon & Lee, 2007). Critical care nurses must consider the implementation of these interventions, since the presence of an active infection in a patient awaiting lung transplantation may result in their temporary removal from the waiting list (Koons & Siebert, 2020).

Patients felt cared for and encouraged when trusting relationships were established with healthcare providers, decreasing feelings of vulnerability. Using consistent and clear communication, compassionately and appropriately in tone and timing, and providing excellent care establishes a trusting relationship between patients and healthcare providers (Knudson et al., 2022).

The establishment of positive and trusting therapeutic relationships with patients is recognized as a fundamental aspect of nursing practice to anticipate care needs, with the potential to significantly impact the quality of care provided (Feo et al., 2017). This requirement aligns with assumptions underlying the Nursing as Caring theory since one of them is that people experience care moment by moment (Boykin & Schoenhofer, 2013). The concepts covered in this nursing theory emphasize the interpersonal relationship between the nurse and the person cared for (Bailey & Wagner, 2009). By intentionally entering the world of the person receiving care, nurses have the purpose of getting to know them as a person who uniquely experiences care, enabling them to express their needs and request care about what matters to them at that moment (Boykin & Schoenhofer, 2013), since vulnerability decreases when care is individualized and personalized (McKinley et al., 2002).

In addition to the establishment of trusting relationships, the physical presence of the patient's family members during a time of great uncertainty and insecure, such as the period in which the person is in the ICU on ECMO while waiting for a lung transplant, decreases negative feelings such as loneliness, fear and anxiety, providing comfort and safety, increasing the peace of mind of the patient (Knudson et al., 2022; McKinley et al., 2002). Likewise, the presence of family plays a vital role in more effective advocacy on behalf of patients. Therefore, nurses, by being present, promoting the presence of family members, and establishing a trusting relationship, contribute to reducing patient vulnerability in the psychological domain and increasing their personal capacity (Knudson et al., 2022; McKinley et al., 2002; Scanlon & Lee, 2007). According to Scanlon and Lee (2007), personal capacity can affect a patient's ability to prevent vulnerability, so an increase in personal capacity can decrease a patient's feeling of vulnerability. Although described the use of advocacy by family members, this is also a strategy that nurses can use to reduce patient vulnerability and increase personal capacity by defending the patient's best interests (Scanlon & Lee, 2007).

Given the above, these findings align with the article by Scanlon and Lee (2007), stating that psychological vulnerability can be avoided through a series of autonomous nursing actions.

Transversal to the three articles that make up this review is the use of awake ECMO as a strategy. This strategy implies a multidisciplinary approach and interventions, including the nursing team, bringing benefits in reducing physical and psychological vulnerability and increasing patients’ resources. This multidisciplinary approach highlights the importance of teamwork, as it is essential for ensuring the quality and safety of healthcare delivery in the ICU, where healthcare professionals with complementary training and skills are committed to achieving shared common goals through a sharing climate and collaboration (Dietz et al., 2014; Oandasan et al., 2006).

The awake ECMO strategy prevents the use of sedation and IMV, favoring communication and the development of a trusting relationship between the patient and the multidisciplinary team (Knudson et al., 2022; Vayvada et al., 2021), as well as the patient's possibility to participate in their clinical progression and interact with family members (Nosotti et al., 2013) at this moment of significant vulnerability. In addition to participation in the decision-making process, Kearns and Hernandez (2016) show that people on awake ECMO have better physical conditions pre-transplant and can also participate in pretransplant education and counseling.

Awake ECMO allows patients to maintain their autonomy and identity, as evidenced in the study of Knudson et al. (2022) enabling a patient to continue working through electronic devices during hospitalization. For him, maintaining as much normality as possible during hospitalization and illness was synonymous with fighting for his life.

Nursing interventions during awake ECMO are emphasized in helping to preserve the satisfaction of fundamental human needs such as oral feeding and hydration and active mobilization (Nosotti et al., 2013; Vayvada et al., 2021), which are essential for effective coping with physical and psychological problems (Vayvada et al., 2021). As Kearns and Hernandez (2016) claim, nurses play a central role in caring for these multifaceted patients, becoming the facilitators of achieving these effective coping strategies by managing and coordinating care throughout the day.

According to Nosotti et al. (2013), awake ECMO is a feasible, effective, and safe strategy in bridge to lung transplantation, preventing patients from deteriorating rapidly during this time and achieving better postoperative survival. Despite claiming the same, Kearns and Hernandez (2016) state that ECMO itself is not without significant risks. However, the awake ECMO strategy performed by a trained multidisciplinary team can be helpful compared with the conventional ECMO strategy (Lee, 2022).

## Limitations

The limitations of this review are due to the size of the bibliographic sample and the heterogeneity of the studies, highlighting the need for more scientific evidence on the subject in nursing. Given this limitation, we included studies in which ECMO was not used as a bridge to lung transplantation, and we adapted the findings for the study population in this review. In addition, the presence of articles written in a language not mastered by the reviewers in the selection process led to its exclusion, preventing the analysis of its relevance to this review.

## Implications for Practice

This review highlighted that, despite the increasing technical complexity associated with critical nursing care, nurses need to delve deeper and become more aware of a person's vulnerability during ECMO as a bridge to lung transplantation, as its presence could affect the health status, bridging process, and outcome. Therefore, the recognition and promotion of expert skills and interventions that minimize vulnerability are paramount for ensuring high-quality patient care. In the field of research, the results of this review could be used to develop a tool for assessing the degree of vulnerability and consequently the effectiveness of implemented nursing interventions. Additionally, the insights from this review can contribute to the implementation of person-centered care measures and policies.

## Conclusion

Vulnerability is experienced by patients uniquely and singularly, being influenced by personal resources and the support environment. The close relationship between both domains of vulnerability is evident, meaning that physical and psychological vulnerability influence each other, exacerbating this condition.

This review's findings allowed for identifying nursing interventions that minimize the person's vulnerability during ECMO as a bridge to lung transplantation in physical and psychological domains. By recognizing and identifying the person's vulnerability during ECMO as a bridge to lung transplantation, nurses can implement effective interventions to minimize vulnerability in this population, thus contributing to the person's well-being through personalization and individualization of care.

With the conclusion of this review, it's recommended that further research and studies with a higher degree of evidence be conducted to identify and support more nursing interventions that minimize a person's vulnerability during ECMO as a bridge to lung transplantation and to gain a better understanding of the experience during the bridging process, due to the scarcity of literature on these topics.
